# Polysaccharide-Rich Fraction of Noni Fruit (*Morinda citrifolia* L.) as Doxorubicin Co-Chemotherapy: Evaluation of Catalase, Macrophages, and TCD8+ Lymphocytes

**DOI:** 10.3797/scipharm.1510-01

**Published:** 2015-03-12

**Authors:** Ediati Sasmito, Triana Hertiani, Tiya Novlita Renggani, Brata Jaya Laksana

**Affiliations:** 1 Laboratory of Medicinal Chemistry, Faculty of Pharmacy, Universitas Gadjah Mada, Sekip Utara, 55281, Yogyakarta, Indonesia; 2 Laboratory of Phytochemistry, Faculty of Pharmacy, Universitas Gadjah Mada, Sekip Utara, 55281, Yogyakarta, Indonesia; 3 Faculty of Pharmacy, Universitas Gadjah Mada, Sekip Utara, 55281, Yogyakarta, Indonesia

**Keywords:** Polysaccharides, Noni fruit juice, Co-chemotherapy, Doxorubicin

## Abstract

Noni fruit (*Morinda citrifolia* L.) has been acknowledged for its cytotoxic and immunostimulatory activity. Our previous results on the immunomodulatory effect of a noni juice polysaccharide-rich fraction encouraged this research to evaluate the potency of the polysaccharide-rich fraction as co-chemotherapy with doxorubicin (DOX) administration. Macrophage activity (MA) was evaluated with the latex bead method. The phagocytic index (PI) was measured as the number of latex beads ingested by 100 macrophages, while the phagocytosis ratio (PR) was indicated by the percentage of macrophages that ingested three or more latex beads. The CEC was evaluated by using a commercial assay kit, while CD8+ T lymphocyte proliferation was evaluated using a flowcytometry method following *in vivo* administration. Thirty male Wistar rats were divided into five groups (n = 6 each). The control group received DOX via i.p. at a concentration of 4.67 mg/kg BW on days 1 and 4; four treatment groups received PF p.o. at a concentration of 25; 50; 100; 200 mg/kg BW daily, respectively, and additionally DOX i.p. 4.67 mg/kg BW (days 1 and 4) for 7 days. The phagocytic activity was not affected significantly by PF administration compared to the Dox control, but PF administration at a dose of 25 and 50 mg/kg BW has been proven to increase TCD8+ cell proliferation in combination with DOX. The catalase concentration, on the other hand, significantly decreased following PF administration at a dose of 100 mg/kg BW. The results suggest that the polysaccharide-rich fraction of noni juice might induce immunomodulatory effects via TCD8+ activation, have antioxidant activity, and thus might be a potential candidate to be used as an adjuvant to DOX chemotherapy.

## Introduction

Doxorubicin (DOX) is the drug of choice in many cancer therapies. Unfortunately, its damaging effects not only occur on cancer cells, but also on normal ones [1]. DOX side effects, such as cardiomyopathy, have been found to be related to the formation of free radicals after reacting with oxygen [2]. DOX can also affect the immune system by decreasing interleukin-2 (IL-2) and producing interferon-γ (INF-γ), decreasing natural killer cells, lymphocyte cells, the CD4+/CD8+ ratio, as well as damaging the thymus organ [3, 4]. Because of these harmful side effects, the use of natural products as a complementary therapy for cancer treatment has been widely accepted. In this case, an immunomodulatory natural product with antioxidative and antiproliferative activity might be beneficial to DOX as a supplemental therapy in cancer treatment.

Noni fruit (*Morinda citrifolia* L., Rubiaceae) has been widely used as alternative medicine with wide therapeutic values, traditionally. Hirazumi and Furusawa (1999) reported that the polysaccharide-rich fraction separated from the noni juice by ethanol could extend the life span of rats implanted with with Lewis lung carcinoma by 75% compared to the control. The polysaccharide-rich fraction could also stimulate the release of several mediators from murine effector cells, including tumour necrosis factor-α (TNF-α), interleukin-1β (IL-1β), IL-10, IL-12 p70, interferon-γ (IFN-γ), and nitric oxide (NO), but had no effect on IL-2 and suppressed IL-4 release. The activities have been correlated to its potential against cancer by stimulating the host’s immune system [5]. Previous research also revealed that the polysaccharide-rich fraction of noni juice (PF) could increase mice’s macrophage activity and lymphocyte proliferation in an *in vitro* assay [6].

Noni fruit juice has also been reported to be a potential antioxidant of which its activity is 2.8 times higher than vitamin C, 1.4 times higher than pycnogenol (PYC), and almost comparable to grapeseed powder [7]. The juice is reported to decrease serum malondialdehyde (MDA), and increase superoxide dismutase (SOD) activity in blood [8]. Catalase, glutathione peroxidase (GPX), and superoxide dismutase (SOD) are the most important enzymes of the cell antioxidant defense system [9]. A clinical trial on heavy cigarette smokers showed reduced DNA oxidative damage following the consumption of 29.5–118 mL of noni juice daily for 1 month [10].

Besides the antioxidant and immunomodulatory activity, noni fruit also has promising anti-cancer properties. Approximately 41% of female patients utilize various types of complementary and alternative medicine (CAM) to manage their breast cancer, including products from the *Morinda citrifolia* plant (noni) [11]. One study showed that Tahitian noni juice may prevent mammary breast cancer at the initiation stage of chemical carcinogenesis [12]. Another study demonstrated the anti-growth effect resulting from the induction of apoptosis, the activated caspase-3 cells in tissues, and decreased proliferation on Ehrlich ascites tumor grown in female Balb-c mice; thus concluding that noni may be useful in the treatment of breast cancer either on its own or in combination with doxorubicin [13].

This research was conducted to investigate the potency of the polysaccharide-rich fraction of noni juice (PF) as a complementary therapy to DOX which will be formulated as *in vitro* and *in vivo* experiments.

## Results and Discussion

The yield of the polysaccharide-rich fraction of noni juice (PF) was 0.003% w/w. The carbohydrate content measured by the phenol-sulfuric acid (PSA) method was determined as 43.056 ± 1.723% w/w. Reducing substances were in an amount of 0.012 ± 0.001% w/w GAE (Folin Ciocalteau Method). Bui *et al*. reported that PF contained galactose units predominantly in the polysaccharide structure. Galactose is one of the reducing sugars which may contribute to the reducing activity [14].

Macrophage activity was not affected significantly by PF administration compared to the baseline (DOX only) as seen by the results of the phagocytic activity (Tables 1 and 2). There was a slight improvement at doses of 25, 50, and 100 mg/kg BW for PF treatment on the phagocytic ratio, but no dose-dependent relationship was observed. These findings certainly showed a contradiction to the previous experiments. Based on Hirazumi and Furusawa, PF effectively enhanced the production of NO, TNF-α, IL-1β, and IL-12 p70 from thioglycollate-elicited adherent peritonial exudate cells, which are mainly composed of macrophages. The same authors also found that PF also enhanced the release of IFN-γ, a potent macrophage activator [15] which may serve as a positive feedback mechanism to promote further activation of macrophages. Further, Setiawan reported an increase in mice’s macrophage activity for PF both for the phagocytic ratio and phagocytic index in an *in vitro* assay [6], which is also in contradiction to our results.

**Tab. 1:**
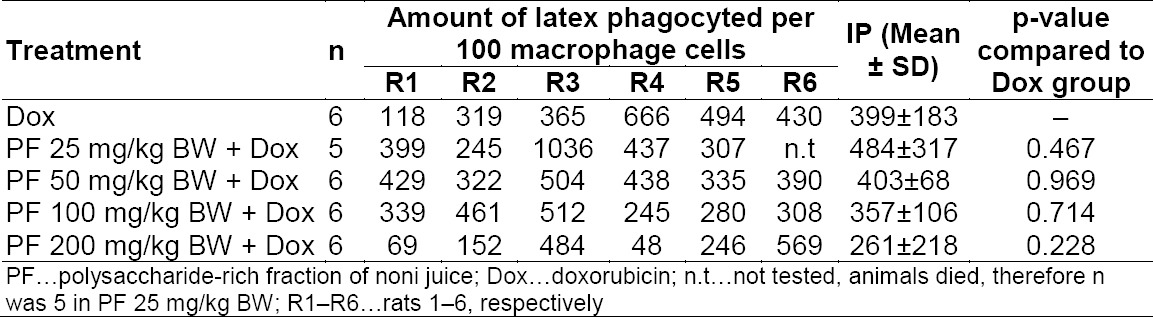
Effect of polysaccharide-rich fraction of noni juice (PF) on phagocytic activity of macrophage cells. Phagocytic activity was determined as the phagocytic index (PI). P-value was determined through one-way ANOVA with α = 0.05

**Tab. 2:**
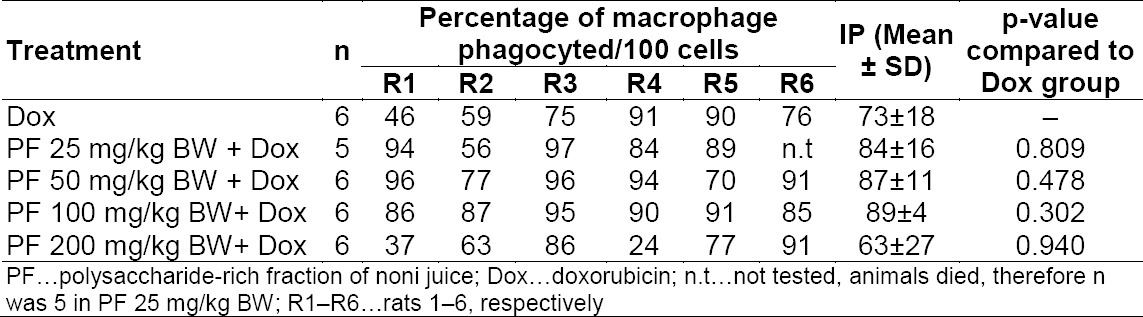
Effect of polysaccharide-rich fraction of noni juice (PF) on phagocytic activity of macrophage cells. Phagocytic activity was determined as the phagocytic ratio (PR). P-value was determined through one-way ANOVA with α = 0.05

As a possible explanation, the immunomodulatory effect of PF *in vivo* may be achieved by a different mechanism rather than via macrophage activation, such as via TCD8+ proliferation, as shown on Table 3. The amount and percentage of TCD8+ proliferation increased significantly after PF administration on doses 25 and 50 mg/kg BW for 7 days. The baseline (Dox only) showed a significant reduction in the TCD8+ profile, which implied that doxorubicin administration supressed TCD8+ proliferation. On the other hand, PF administration at a dose of 200 mg/kg BW caused a significant decrease in TCD8+ proliferation. However, an explanation of the unexpected results shown by the high doses of PF administration (100 and 200 mg/kg BW) was not revealed yet.

**Tab. 3:**
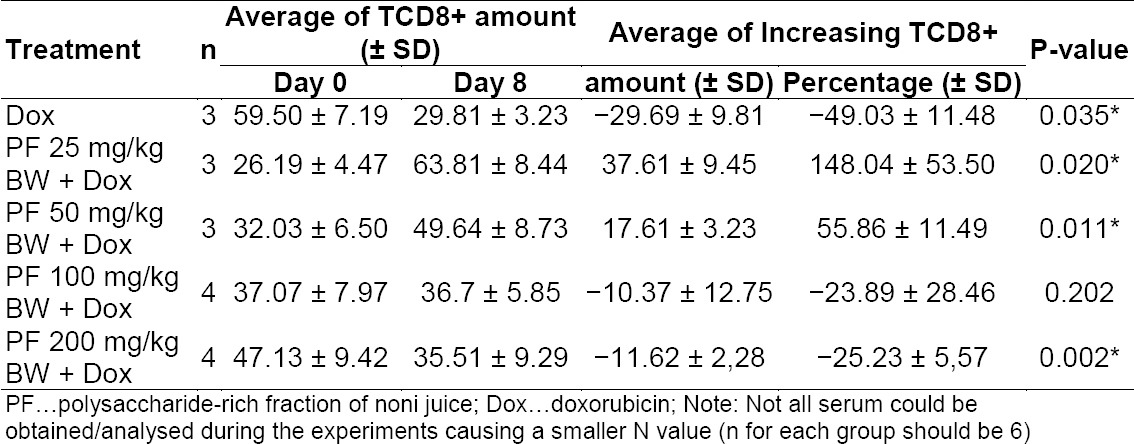
Amount of TCD8+ before (day 0) and after treatment (day 8). P-value was determined through the paired t-test with α = 0.05. Asterisk signs indicate results that were significantly different from the DOX-only treatment (P<0.05)

The influence of PF on the cell-mediated cytotoxicity T cells (TCD8+) may be inferred from its suppressive effect on IL-4 production, as well as its stimulatory effect on IL-12 p70 production. IL-4 and IL-12 generally have opposing effects on cell-mediated immunity [16]. IL-4 acts in most instances oppositely to the Th1 cellular immunity response, whereas IL-12 promotes a Th1 response. The contrary effects of PF on IL-4 and IL-12 production may augment the Th1 response and enhance the cytotoxicity of CTL (TCD8+). Further research done by Hirazumi and Furusawa also showed that the addition of exogenous IFN-γ, a Th1 cytokine, enhanced the antitumour activity of PF [17]. IFN-γ is the final end product of the Th1 response and it directly activates cytotoxic T lymphocytes (TCD8+), NK cells, and macrophages. Consequently, the activated TCD8+ and NK cells release more IFN-γ, which itself is cytotoxic and stimulates tumour cells to express Fas. Further, this transmembrane protein triggers apoptosis when bound to the Fas ligand, of which is expressed only on the activated NK and T cells [18, 19]. These findings also support previous research done by Setiawan that PF increased mice’s lymphocyte proliferation in an *in vitro* assay [6].

As one of the antioxidative-related enzymes, the catalase concentration increased in the case of elevated oxidative stress resulting from lipid and protein damage [20]. The results demonstrated that in every group, the catalase concentration decreased (Table 4), but the reduction was not statistically significant, except for the 100 mg/kg BW group. Consistent with the result in TCD8+ increase, the reduction in catalase concentration was higher in 25 and 50 mg/kg BW groups in comparison to the higher dose application. Nevertheless, no dose-dependent relationship was observed in accordance to the reduction of catalase concentration. These findings suggested that the polysaccharide-rich fraction from noni juice also had antioxidant activity, despite there being no clear evidence yet.

**Tab. 4:**
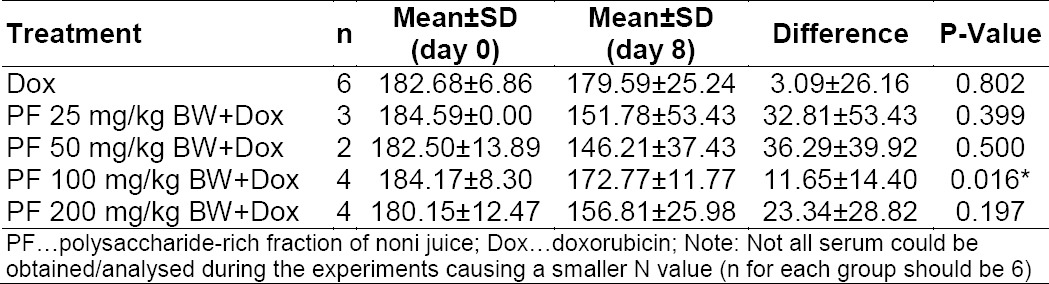
Difference of catalase titer before and after treatment with polysaccharide-rich fraction of noni juice (PF). Catalase concentration stated in U/mL. P-value was determined through the paired t-test with α = 0.05. Asterisk signs indicate results that are significantly different compared to DOX-only treatment (P<0.05)

As a conclusion of this study, the polysaccharide–rich fraction of noni juice may be a potential candidate to be used as an adjuvant to DOX chemotherapy in order to reduce its side effects.

## Experimental

### Sample Preparation

Ripe noni fruits were collected from Sleman, Yogyakarta in 2013. Taxonomy identification was performed in the Pharmaceutical Biology Department, Faculty of Pharmacy, Universitas Gadjah Mada, Indonesia, of which the plant was grown in the faculty garden. The certificate was issued under Nr. BF/19/Ident/Det/II/2014.

The fractionation of the polysaccharide-rich fraction of noni juice was prepared according to Furusawa’s method [17] with a slight modification [6]. The juice was collected by keeping the clean fruit inside a closed jar protected from sunlight for 3 days. The liquid that seeped out of the fruit was collected and 96% ethanol was added to separate the polysaccharides. The sediment rich in polysaccharides was then centrifuged at 3,500 rpm for 35 min. The sediment obtained from centrifugation was rinsed with ethanol and freeze-dried to yield the polysaccharide-rich fraction (PF) in a light-brownish powder form.

### Animal Treatment

As many as 30 male Wistar rats, 10–12 weeks old having a weight range of 150–200 g, were used for the experiments. They were obtained from a local breeding UD Wistar certified by the Head Laboratory of Faculty of Veterinary, UGM, Indonesia. T47D and Vero cells were obtained from the Integrative Research and Testing Laboratory, UGM, Indonesia. Five groups consisting of six rats each were placed in cages for 7 days for conditioning. Food and water were given *ad libitum*. Husk was used to absorb urine and was replaced every 3 days. The animal protocol and ethics of the institute were strictly adhered to for the international principle of animal handling guideline procedures [21]. Groups were divided into a negative control (doxorubicin i.p. 4.67 mg/kg BW on days 1 and 4); 25; 50; 100; and 200 mg/kg BW of PF p.o. daily (from days 1–7); and doxorubicin i.p. 4.67 mg/kg BW (days 1 and 4).

Male Wistar rats were used in order to minimize bias due to influences of hormonal changes which might be found to a greater extent in female rats.

### Catalase Enzyme Assay

One mL blood was taken from the rats’ plexus retro-orbitalis on day 0 (pre-treatment) and 8 (post-treatment), left at room temperature for 30 min to form a clot, then centrifuged in 4°C, 4,000 rpm for 10 min to separate the serum. The assay was using the Amplex Red Catalase Assay Kit A22180 (Invitrogen) and was performed according to the standard procedure of the assay provided with the kit.

### Macrophage Activation Assay

Macrophages were isolated from Wistar rat peritoneal fluid by adding 10 mL of cold RPMI 1640. The aliquot was centrifuged at 1,200 rpm 4°C for 10 min. After we decanted the supernatant, about 3 mL of RPMI 1640 complete media (containing FBS 10% (v/v)) was added to the sediment clumps. The cells were counted by the haemocytometer and then resuspended in a complete medium to obtain the cell suspension. The suspension was then inoculated on 24-well microtiter plates which were covered by round cover slips. Each well filled with a 200 μL suspension contained 2.5×10^5^ cells. Incubation took place in a 5% CO_2_ incubator at 37°C for 30 min. Afterwards, 1 mL of the complete medium was added into each well and reincubated for another 2 h. After rinsing twice with RPMI 1640, 1 mL of complete media was added, followed by 24 h incubation. Phagocytic activity measurements were performed by using 3 μm latex beads resuspended in PBS to get a concentration of 2.5 × 10^6^ mL^−1^. The 24 h-cultured peritoneal macrophages were washed twice with RPMI 1640. The latex suspension (200 μL/wells) and samples (200 μL/wells) were added. The suspensions were then incubated in a 5% CO_2_ incubator at 37°C for 60 min. The cells were washed three times with PBS to eliminate excess latex beads. After drying at room temperature, fixation was done with methanol for 30 sec. Afterwards, the methanol was aspired and cover slips were left to dry, followed by 2% Giemsa (v/v) staining for 20 min. After being washed with distilled water, the cover slips were left to dry at room temperature.

The amount of macrophages which phagocyted the latex beads, as well as the amount of latex beads consumed by the macrophages, were counted under an inverted microscope to calculate the macrophage phagocytic index and ratio.

### Lymphocyte Proliferation Assay

Fifty µL of the blood sample from each group were placed into a reagent tube and 10 µL of monoclonal antibody PE Rat Anti-Mouse CD8 and FITC Rat Anti-Mouse CD3 were added. The mixture was vortexed, followed by 15 min incubation in a dark place. Afterwards, 450 µL FACS lysing solution was added and the mixture was incubated for 10 min in dark. Furthermore, centrifugation took place at 1,500 rpm for 5 min. The sediment was added with 300 mL PBS buffer. Cells were transferred into a cuvette and placed under the flowcytometer nozzle. BD Cell Quest Pro™ was used to obtain data in the form of a relative amount of lymphocyte TCD8+.

### Data Analysis

The data obtained were analysed by using the software SPSS 19 at a 95% confidence level.

### Phytochemical Analyses

Several planar chromatography systems were evaluated to get the best separation of the active fraction’s chemical contents. Toyo paper Nr. 1 was used as the stationary phase, n-buthanol : glacial acetic acid : water (6:1:2 v/v) as the mobile phase, and KMnO_4_ spraying was used to detect the reducing sugars. Glucose was used as the standard. Furthermore, to detect the presence of other chemical contents, a TLC system having silica gel F 254 as the stationary phase and a mixture of ethyl acetate : n-propanol : glacial acetic acid : water (4:2:2:1 v/v) as the mobile phase were chosen. UV 254 and 366 nm lamps were used to detect the compounds with chromophores. Spray reagents i.e., FeCl_3_ and anisealdehyde-sulphuric acid were used to detect the chemical content groups of the compound [22].

Following KMnO_4_ spraying, the spot of the hydrolyzed sample showed an Rx value of 1.00 (compared to a glucose standard), of which the non-hydrolyzed sample showed a lower value of Rx 0.68 (Toyo paper Nr. 1, n-buthanol : glacial acetic acid : water (6:1:2 v/v)).

FeCl_3_ spraying on the eluted sample showed a negative result, suggesting that no compound having free phenol groups was contained in the sample. On the other hand, the same TLC system that was used detected the presence of saponin after being sprayed with the aniseldehyde-H_2_SO_4_ spraying reagent. The spot in the sample showed a similar pattern with the saponin standard used (Rx value of 1.00). This result was supported by a positive result of the tube test run for the saponification ability of the samples.

### Determination of Total Phenolic Content

Total phenolic content of the extract and fractions was determined by the Folin-Ciocalteu method [23]. Certain volumes of the sample and standard solutions (1 mg/mL) were oxidized with 0.4 mL Folin-Ciocalteu reagent. After being left for 5 min, the solutions were neutralized by the addition of 4 mL 7.5% Na_2_CO_3_. Following 120 min incubation at room temperature, absorbances were measured at 760 nm. Total phenolic contents were calculated as gallic acid. Considering that neither phenolics nor protein were detected by the above phytochemical experiments, this suggests that the reducing substance in the PF was reducing sugars.

### Determination of Total Polysaccharide Content

Total polysaccharide contents of the extract and fractions were determined by the AOAC [24]. Briefly, 150 mg dextran (standard) was hydrolyzed with 25 mL HCl 2 N and 25 mL aquadest at 100°C for 2 hours. The hydrolyzed solution was placed in the 100-mL volumetric flask, to which distilled water was added to reach the 100 mL mark. The standard solution was prepared by taking a certain volume of stock solution and then adding 200 mL of 5% v/v phenol in water. Concentrated H_2_SO_4_ (1 mL) was rapidly added to the mixture and incubated for 10 min at room temperature, followed by incubation for 15 min at 37°C. Absorbances were measured at 490 nm using a UV-VIS spectrophotometer. The polysaccharide fraction (PF) was treated accordingly.
